# Educators Perspectives on the Value of Physical Education, Physical Activity and Fundamental Movement Skills for Early Years Foundation Stage Children in England

**DOI:** 10.3390/children8050338

**Published:** 2021-04-26

**Authors:** Alexandra Dobell, Andy Pringle, Mark A. Faghy, Clare M. P. Roscoe

**Affiliations:** Human Sciences Research Centre, College of Science and Engineering, University of Derby, Kedleston Road, Derby DE22 1GB, UK; a.pringle@derby.ac.uk (A.P.); m.faghy@derby.ac.uk (M.A.F.)

**Keywords:** physical activity, physical education, early years foundation stage, educators

## Abstract

There is a lack of information available for physical education (PE) provision in the early years foundation stage (EYFS), prompting concern about what is currently delivered in schools and the values behind the approaches taken. Using semi-structured interviews, this study investigated educators’ perspectives on the value of PE and physical activity (PA) for EYFS children across England in relation to opportunities for, barriers to, and benefits of PA and PE. This study collected important stakeholder views and can help shape the impact and implementation of fundamental movement skills (FMS) and PA interventions at the EYFS.

## 1. Introduction

The UK Chief Medical Officer’s guidelines for children aged 5 years and under state that children should achieve at least 180 min of physical activity (PA) every day, with 60 min of this categorised as moderate-to-vigorous PA (MVPA) [1]. Current reports show that 46.8% of all school aged children are reaching “active” levels in England [2]. Further to this, one in ten children start school obese, and a further 13% as overweight, with these figures rising to 35% of children overweight or obese at ages 10 to 11 years old [3]. Evidence demonstrates the link between low PA and high levels of sedentary behaviour increasing obesity risk for children [4], and, importantly, there is a need for meaningful PA to be implemented into children’s daily lives.

Educators play important roles in ensuring children are physically active whilst at school. PA is vital for physical, mental and social well-being in children, reducing the likelihood of disease and illness [5,6]. During the early years foundation stage (EYFS) (4 to 5 years old), physical education (PE) and PA opportunities are also influential in developing and building children’s fundamental movement skills (FMSs). FMSs are the building blocks to more complex movement patterns which are made up of object control (e.g., throwing and catching), locomotor (e.g., running and jumping), and stability skills (balance and flexibility) [7]. FMSs competency contributes to children’s gross motor development, which is associated with sustained PA levels in later life [8,9,10]. Therefore, early years PE and PA opportunities should allow children to practice and develop their FMSs, to develop healthier lifestyle behaviour trajectories and reach school specific PA guidelines of 30 min of MVPA per day [11].

Duncan and colleagues found that children aged 6–9 years old attending school in England are not competent in basic FMSs [12]. In this age range, children are taking part in more sport opportunities and performing PA in game environments, where competency in these skills is expected in line with the educational curriculum [13]. Gallahue, Ozmun, and Goodway [14] proposed the concept that children can be potentially proficient in all FMSs components by the age of 6 years. Accordingly, consideration to tuition, practice and development of FMSs should be introduced at early childhood stages to see this competency increase. Settings are a pivotal consideration when planning interventions [15]. Schools are important socialising agents that can facilitate increased PA and should be considered as critical environments for developing FMSs [16,17].

Chronic underfunding in education over the past 10 years has seen access to PE in English schools reduce dramatically [18]. Moreover, the Institute for Fiscal Studies reported an 8% decrease in spending per pupil between 2009/10 to 2019/20, due to higher class sizes and reduced teacher numbers, resulting in lower-quality education [19]. Although primary PE and sport premium funding has increased during this time [20], there is still a decreased commitment to provide more or better quality PE provision within school environments, with a continued increased emphasis on improving academic attainment [21]. An important consideration is that the EYFS is only provided with a statutory framework. Physical development of co-ordination, control, and movement is highlighted; however, the framework lacks further depth around what this may include for PA opportunities and PE. Indeed, the UK government does not provide a curriculum for PE before Key Stage 1 (KS1), which begins at 5 years of age in England [22], leaving individual schools and teachers to select the provision for the EYFS, despite FMSs and PA being key to children’s physical development, and, gross and fine motor development. This potentially renders teachers inadequately equipped in knowledge, time, and resources to deliver high quality PE and achieve FMSs mastery.

Considering Newell’s model of dynamic constraints [23], the individual, environmental and task constraints can affect movement development, and thus the achievement and attainment of FMSs mastery. Stodden’s 2008 [4] dynamic association model demonstrates that higher FMSs competency can increase PA in children. Further to this, factors that influence PA levels that children achieve are well established [24]. Notably, children from minority ethnic backgrounds, those with disabilities or long-term health conditions, and living in areas of higher deprivation commonly report low PA levels throughout England [2]. Sex has also been repeatedly reported to influence FMS competency at the early years [9,25], as females have tended to show higher locomotor abilities than their male peers, and males are more competent in their object-control abilities than females. This is despite little biological differences between boys and girls at these ages. In beginning to address these determinants, increasing educator’s knowledge, and resources, to deliver PE and PA that is developmentally appropriate for all children will lead to improved outcomes for children’s all-round health and habitual PA levels [26].

The diverse determinants impacting delivery and public health guidance on intervention design highlights the importance of involving key stakeholders in the development and implementation phases [15]. However, there is a lack of research involving EYFS teachers, head teachers, and PE specialists [27]. They can provide rich insights into the practicalities of delivering interventions and this intelligence can shape PA provision [28]. To the best of our knowledge, there is a lack of qualitative inquiry investigating key influencers of PE, PA, and FMSs in English schools. Heads of schools, EYFS teachers, and PE educators have been identified as key influencers of PA in National Public Health guidance [29]. Accordingly, the aim of this study was to capture the perspectives of these educators on the value of PA, PE, and FMSs in school settings for the EYFS, including views on impact and implementation.

## 2. Materials and Methods

### 2.1. Study Design

The current study was underpinned by a constructivist philosophy and explored perspectives and views through qualitative research methods and study design. Semi-structured individual interviews of a phenomenological approach were conducted to examine the interviewees experiences, perspectives, values, and opinions of the topic in question [30]; these opinions considered how FMSs and PA are delivered in EYFS settings. Focusing on the empirical world for the educators was important in deriving the real-world experience such as the “how” and “what”, not just their “why” for the different approaches and methods they took with their PE and PA delivery.

### 2.2. Participants

Following ethics approval from the host University (ETH1920-2939) and informed consent, a sample of educators responsible for the delivery of EYFS, PE, and PA participated (*n* = 12 consisted of head teachers *n* = 2, EYFS teachers *n* = 7, and external PE providers *n* = 3). Participants were recruited from primary education settings around England through professional connections, word of mouth and social media channels. The definition of educators recruited within this study included educators with a role that dealt with (or had recently dealt with) the organisation and/or delivery of PE for EYFS children in the UK.

The sample included a heterogeneous sample of participants (females, *n* = 9), with a homogenous age range, experience in primary education ranged from 2 years to over 30 years. There was also a homogenous sample of socioeconomic spread (SES) which can be viewed in Appendix A (Table A1), the English indices of deprivation (IoD) [31] was used to assess deprivation of schools. The IoD assess seven different areas or domains affecting levels of deprivation, including income, employment, health deprivation, education skills and training attainment, barriers to housing and services, living environment quality, and crime levels [31]. The areas are rated from 1 (the most deprived) to 32,844 (the least deprived).

### 2.3. Instrumentation

Single semi-structured individual interviews of around 20 to 45 min were used to allow for a sufficient conversation to occur between the interviewer and interviewee; some participants provided higher levels of detail than others. Semi-structured interviews allow for reliable, detailed, and comparable data to be collected [32], in addition to the interviewees’ full views to be expressed and voiced. Questions were developed by using the literature [12,22,33,34], prior knowledge [25] and experience of FMSs and PA development in young children. The decision to use not only EYFS teachers but also head teachers and external PE providers was important, since, in modern schooling within England, these can play an influential role in child development, including FMSs ability, as well as the levels of PA children achieve in their school day. An example of an external provider would be a coach or PE specialist who exclusively delivers PE in school and may do so across multiple locations. Their use has increased in recent years due to the PE and sport premium funding provided to English primary school settings [20]. Schools use their expertise to support and develop primary teacher’s PE delivery.

Although planned as face-to-face interviews, COVID-19 restrictions meant interviews were conducted remotely on the software program Zoom. This allowed views and perceptions to be collected nationwide and avoided the clustering of opinion in one area. Conducting interviews remotely also allowed participants to be in a familiar environment and feel at ease, whilst allowing flexibility of when to attend their interview, overall resulting in better outcomes [35]. The interviews were recorded, downloaded, and transcribed verbatim.

### 2.4. Researchers

The interviews were conducted by a single trained interviewer (the first author, A.D.).

Piloting of the questions was followed by a process of refinement to ensure the questions were relevant to achieve desired information and allow extended answers by the participant. The interviewer also openly asked questions not within the schedule to allow participants elaborate on interesting points made. At all stages of the research process, regular group and individual researcher discussions were held, assuring the quality of the research processes and confirming the trustworthiness of the data.

### 2.5. Procedure

Four main topics of question were used during the interviews. These were centred on PA and PE delivery within schools. The questions were used to assess (1) opportunities and determinants for quality PE, PA, and FMSs practice and delivery in schools; (2) how PA and PE affect children both positively and negatively; (3) educator confidence and knowledge to deliver PA and FMSs interventions to EYFS students within their school settings; and (4) values and feelings towards FMS, PA, and balance importance for young children. The topic areas and questions can be viewed in Appendix B. Open ended questions ensured that the full views, perspectives and knowledge could be gained from the educator’s answers. The aims of asking these questions and gaining answers was to firstly, fully understand how PE and PA are currently delivered in schools for young children and the experience of educators delivering this. Secondly, to help inform the design, training, and delivery of future interventions for use in educational settings. Finally, these data can be combined with other datasets focussed on the experience children receive in English education settings in combination with quantitative statistics around PA achievement and FMSs mastery. Collectively, these datasets can help inform future policy and intervention from the perspective of a key stakeholder and provide a comprehensive understanding of the areas investigated.

### 2.6. Analysis of Qualitative Data

**Figure A1:**
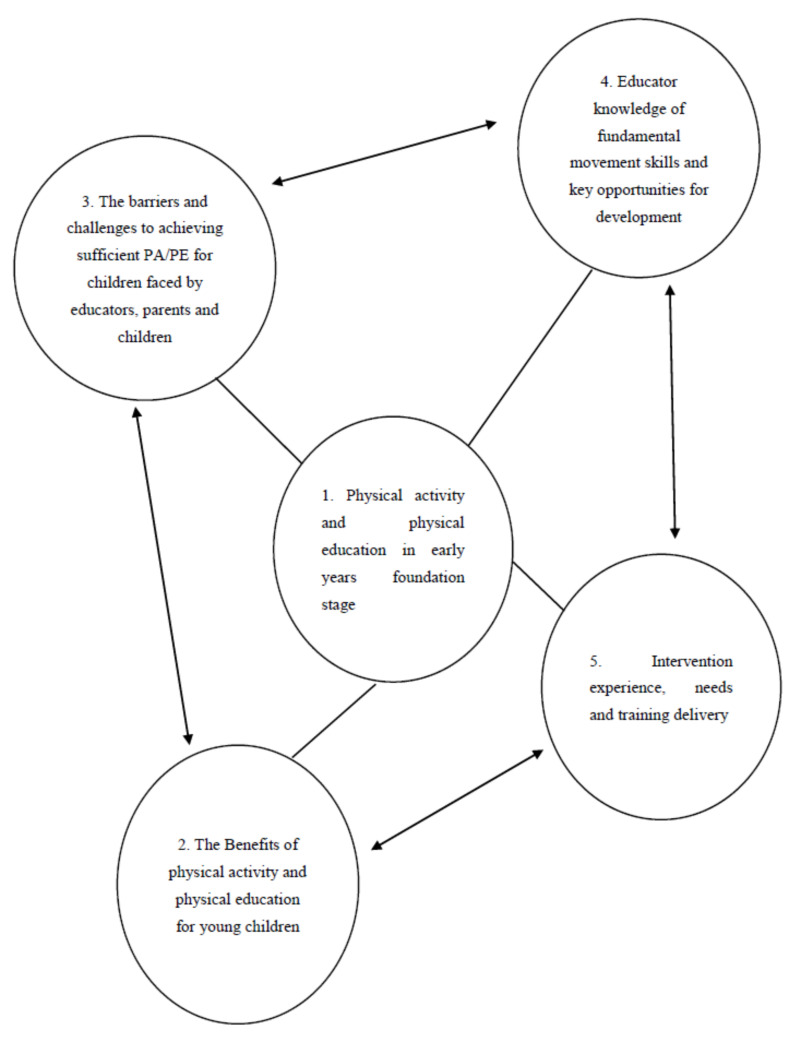
Theme map.

Thematic analysis was used to analyse transcripts, using the six-phase process proposed by Braun and Clarke [36], which has been widely used in PA research [37,38,39,40]. Transcripts were checked for accuracy and reading of the interviews to saturation, they were subsequently coded. Codes identified interesting points and features emerging from the interviewees within the datasets that related the research question and topics surrounding this. Subsequent sense checking between, Alexandra Dobell and Andy Pringle was completed after coding of the transcripts. Codes were grouped into themes, if an extract was coded twice, it was important that both codes sat within the same theme. Following the recommendation of Ryan and Bernard [41], the themes were identified via repetition in topics and answers; similarities and differences in answers to the same question; reflection of the missing data within this research area; theory relating to the scientific underpinning of the questions; and, finally, the metaphors and analogies interviewees used within their answers. AD and AP refined the specifics of the themes to generate clear definitions and names, using the guidance of Braun and Clarke [36]. Initial data and themes were shared independently with the research group to confirm credibility and trustworthiness (CMPR and MAF) and following consensus that the data supported the initial themes, a final set of themes were established. These five themes were used to organise the results (Appendix C, Figure A1).

## 3. Results and Discussion

This study provides an overview of an educators’ perspective on the value of PA, PE, and FMSs in school settings for the EYFS, including views on impact and implementation. No study to date appears to have considered a qualitative view of these factors in England, making a novel contribution to the literature. The current literature tends to focus on PE for pupils at key stage 1 and above, failing to acknowledge the importance of how PA and PE could be delivered in the EYFS.

The results are presented by using a selection of extracts within the text and are summarised in Table 1. Quotes are referenced using pseudonyms for each participant followed by (H) for head teacher, (T) for EYFS teacher, or (E) for external PE provider.

### 3.1. Physical Activity and Physical Education in School Settings for EYFS

All participants described experiences related to PA and PE opportunities in schools. Children in the EYFS “have a lot of play time in reception” Steven (T), they have “break times, lunchtimes, and PE and play opportunities” Jonny (E), “they’ve got lots of equipment out to be using as well” Leah (T) and “In our school we have five-minute movements, between each lesson we’ll do some form of dance, PE, go outside, go for a run” Sarah (T). We have a “PE specialist who works with the children…but that’s only for an hour a week” Melissa (T), while “over the last few years it’s been a gripe of mine that we haven’t got two hours of PE, but I finally won that battle…now we’ve got targeted practice for two hours a week” Lorna (T). These responses demonstrate that schools do have enough time to deliver PA and PE; therefore, it is a matter of getting children to be active through improved promotion of PA rather than extending the time for PA.

Educators highlighted the large amount of free-play children have in the EYFS: “There’s lots and lots of opportunities for outdoor play, and there’s equipment that’s rotated around the week” Lorna (T). In the “early years it’s a lot of learning through play, but structured play” Kristen (T). There was recognition of children accessing outdoor environments and its likelihood of increasing PA engagement: The “behaviour benefits of [outdoor] physical activity” Jonny (E), are immense after “a week of wet play [the children] are agitated” Sarah (T) and “as much as a sports hall is great, the fact physical activity gets you outside, vitamin D, being in the fresh air, it’s so good for the kids” Kristen (T). “They do forest school with me, moving around the forest, think[ing] about how we’re moving” Leah (T). Play opportunities are of great importance in the early years and research has continually found outdoor settings to correlate with higher levels of PA engagement [42].

The Daily Mile or a similar initiative was mentioned by Lorna (T), Leo (E), Melissa (T) and Kristen (T). The UK government actively encourage schools to include an active mile initiative as part of PA provision for children attending primary school, the popularity of this initiative and effectiveness of government PA guidance was observed in the current study [20]. Benefits of The Daily Mile include breaks from classroom environments that can be perceived as pressurised, to increases in MVPA and fitness levels [43,44]. However, “just sticking a daily mile in doesn’t mean they’re developing all the fundamental skills that they need, obviously it helps with being active. But having [skill] focussed lessons, I think will see it improve even more” Lorna (T). This demonstrates educator awareness of the importance of the quality of the PA and PE experiences that children received, with “PE all the way through the school…rather than it being sport related, it is skills related” Brigit (H).

Educators covered areas including the provision of extra-curricular activities for children to achieve more PA that are commonly provided free of charge in English state schools: “We want to get 100% of the kids in each class to take part in at least one club and do one physical activity…we’ve got 89% of children taking part in at least one club” Steven (T), with “numerous different football clubs for boys and girls, multisport clubs, dance, and gymnastics that happen after school” Caitlin (T), and “they have two multi-skills clubs a week, they have ball skills club, they have rugby tots. So, they have an abundance of activities” Kristen (T). Ensuring adequate opportunity is available is a role schools take seriously, and the recognition of children achieving PA guidelines is important. Nonetheless, social norms have led to skills not being developed by girls and boys in the early years, due to the underrepresentation of the skill within a sex’s stereotypical play opportunities [45,46]. Therefore, appealing to both sexes and reducing sex discrepancies in achievement of both FMSs development and PA levels, where sex differences consistently appear [9,47] must be addressed. Establishing interventions that allow developmentally appropriate activities for all may be a crucial step in the EYFS. Before and after school provision is an easy way for both parents and educators to ensure that children are striving to reach PA guidelines. However, not all children will want, or be able, to take part in these clubs as they can in PE lessons, and schools’ resources only stretch so far. Therefore, schools must be considerate of a child’s circumstances to ensure PA guidelines are met within school time [11], and so that the benefits of PA can be obtained by the children in the EYFS.

### 3.2. Benefits of PE and PA for Young Children

Sport, PA, and PE are seen as opportunities for children to strive towards achievements and building intrinsic motivation. Children “have a good self-esteem, so just being able to do those fundamental movement skills, it gives them so much confidence, so that they feel competent in something” Kristen (T) and “developing those personal skills, like perseverance… self-motivation…skill development [and gaining a] sense of accomplishment” Jonny (E), the children “see that they do develop, like being able to achieve new targets and pushing themselves” Leo (E). “I think it is individual goals, and it’s not all about winning, it’s kind of like getting your achievements and making the best out of it” Ruth (E). Further benefits include helping children to “have better social skills, [so] they can deal with conflict a lot better…and failure as well” Caitlin (T). Additionally, “Building a community” Leah (T) and “working together, collaboration, and cooperation…show them that actually you’re only as strong as your weakest link” Brigit (H), and “for me it’s so valuable for your communication skills, your leadership skills” Kristen (T). As well as opportunities to excel outside the classroom with “children who struggle with lots of things, however, can do any physical activity you throw at them” Leah (T), “because there’s more to you than the calculations you can do in your head or how well you can speak a different language” Melissa (T). Early years PE and PA provide an arena for children to develop important skills. A child learning about failure and approaching a task in a different way is key in developing self-management skills, which are important for undertaking regular PA. Traditionally, PE and sports are perceived as broadly physical pursuits for the purpose and improvement of physical abilities and processes. However, PE is more than “sport” to children, demonstrated by the variety of social, communication, and academic skills aforementioned. This fits within the EYFS framework early learning goals of personal, social, and emotional development [22]; therefore, PE and PA must hold an important part in day-to-day EYFS settings. It is important that lesson design considers the opportunity for FMSs skills to be learnt, developed and progressed. Fostering the competence and enjoyment of PA through strong FMSs at an early age will engage children in further PA opportunities though to adulthood [4]. Several educators also related their answers to physical fitness, well-being and health; “sport…it’s everything, it’s the social, it’s the health and well-being, it’s the physical” Brigit(H), “cardiovascular fitness, building muscle strength” Leah (T), “it’s good for your body, keeping your body fit, eating healthy, your mind [and] your brain” Ruth (E). “I was talking to a boy in reception the other day and he was telling me about his heart beating faster, so they become more knowledgeable from having physical activity” Leo(E). In England, the Office for Standard in Education, Children’s Services and Skills (Ofsted) inspect educational settings including schools. Schools hold a responsibility to educate their pupils about leading a healthy lifestyle. For the EYFS, this is assessed by Ofsted, under “personal development”, and their education providing them with knowledge to keep physically and mentally healthy [48]. We propose that the teaching and development of FMSs for children at the EYFS should also be considered to be part of the assessment, due to the positive associations between FMSs competency, physical activity [9], and academic achievement [49], encompassing the purpose of the school environment.

There was recognition of the importance and joint responsibility of the education environment to help tackle the obesity crisis. PE and PA “helps us to reduce obesity and obesity is a ridiculously growing problem, in this country, and I can only see that getting worse” Karis (H). FMSs competency has been previously shown to be a predictor of weight status in children [50], strengthening the argument for high quality PE within schools that fosters FMSs and enjoyment of PA for continued participation.

### 3.3. The Barriers and Challenges to Achieving Sufficient PA/PE for Children Faced by Educators, Parents, and Children

Educators expressed concern about the amount of time that is dedicated to PE planning: “PE is one of the lessons that teachers plan the least…I would say the differentiation is non-existent in PE” Brigit (H). Physical education specialists in England are trained to teach from the beginning of key stage 1. This approach to training PE specialists ignores ages 4 to 5 years old, despite attendance at school being commonplace for these children. “Unless they’ve [EYFS teachers] got a PE background they lack the confidence to teach something like that (PE)” Caitlin (T), and “we had like four weeks training on how to do set PE lessons, not really enough for how important it is” Leah (T). Notably, educators mentioned the lack of confidence to teach specific skills or having the knowledge to implement play-based learning for the development of FMS: “a lot of teachers shy away from it, they’ll put children straight into a game, before they’re ready…without any basic throwing or catching skills” Brigit (H), with a danger of providing poor instruction. The previous literature supports findings that teachers may have good or increased intention to promote PA but lack the resource, knowledge or self-efficacy to appropriately deliver this [51]. Although time is dedicated in the weekly timetable to delivery of PE and PA, it seems largely left to chance of what the content of these sessions might be, especially when a PE specialist or external coach is not involved. Increases in Government funding for primary schools in England, the PE premium, and guidance by the government to invest in further professional development and training for their EYFS teachers, or qualified sports coaches, and PE specialists, to work alongside staff to enhance delivery should enable educators to feel better prepared [20,52].

Barriers to children achieving sufficient PA and opportunities for children to develop FMSs and their gross motor development were highlighted by the educators. This included the recognition of the increasingly sedentary lifestyle that both adults and children lead: “Children [have] more access to tablets, computers, etc. I think culturally things have changed in terms of playing outside” Jonny (E), “kids have maybe got a tablet, and you don’t move on a tablet, do you? It’s just the way people parent has changed hasn’t it, because life changed” Leah (T). There was awareness of the different home environments the children come from, making school environments a key place for PA attainment and FMSs development. “We need more support from out of school. I don’t think it’s put across to parents how important physical education is” Steven (T). “I think it [FMS] probably needs teaching in the schools to be quite honest because, as I said, you don’t always have the enthusiastic parents that want to teach them” Ruth (E), and “sometimes your kids that do enjoy it [PE] don’t get the opportunities when they go home” Steven (T). Educators believed that children that came from more active homes, “your stereotypical group who have got really sporty parents” Leah (T) encouraged further PA opportunities, were likely to “be more physically able” Leah (T). These views were supported across the literature [53,54,55,56], where positive associations between parental activity levels, encouragement, and weight status were consistently observed. A challenge for future research is developing interventions of an informational approach to encourage increased PA in both children and parents.

The current study collected views from educators across socioeconomic spread (Table A1). Deprivation was mentioned as a barrier to children partaking in PA: “less children at my school where I am now take part in extracurricular sporting activities” Leah (T); parents “can’t afford to take them to do extra-curricular things, so they tend to sit in front of a TV all of the time…even just going for a walk and climbing, things like that, they don’t do” Caitlin (T). There was clear awareness of the effect of deprivation: “80% of our children live in the poorest 20% of postcodes in the UK, and 60% have pupil premium… and a lot of the parents have got lots of fears about going outside” Melissa (T). These points highlight the need for further parental education about the PA benefits that can be achieved with few resources and little cost, especially for those from areas of deprivation.

The National Child Measurement Programme [3] statistics for England show that children aged 4 to 5 years old from areas of high deprivation are more than twice as likely to be obese compared to their peers from areas of low deprivation: “I know that we have in reception…a height and weight check, and last year my cohort, somewhere between 20 and 25 [percent] of the cohort were classed as overweight or obese at the age of 4 and 5. Which obviously, is a really shocking statistic that a quarter of the class are overweight” Melissa (T). Although these figures are a cause for concern, it is encouraging that educators have noticed changes in the context of the broader issues and challenges society faces. Prioritising movement and PA opportunities in schools for children, especially those of low deprivation where out-of-school opportunities may be much lower, is essential to seeing improved statistics.

Although deprivation is linked to lower levels of PA in English school children [2], one educator mentioned how some families of higher affluence, commit exclusively to academic rigour and this can negatively influence how parents value PE and PA for children during their education: “She turns around to me and says, ‘my mum says I don’t need friends and I don’t need sport to get into Oxford’” Kristen (T). A head teacher also commented on common behaviours of parents from areas of higher affluence: “It’s lots of parents who really want to do the best thing for their child, but they also won’t do anything that upsets their child. So, it’s… about educating and promoting, but not making parents feel bad about it” Karis (H). Where parents can give children choice about being active, children are choosing to be less active due to the changes within their environment [57]. This shows a need to provide adequate school opportunities that engage children, and educational interventions for both children and their parents across the SES. A review by Nguyen et al., in 2016 found that psychosocial support of parents in PA interventions for children were highly important to intervention success. Additional support from parents would aid children’s FMS, PA and ultimately their academic achievement.

### 3.4. Educator Knowledge of Fundamental Movement Skills and Key Opportunities for Development

Overall, educators showed some knowledge regarding the basis of FMSs. Running, jumping, hopping, throwing, skipping, and catching were skills that eight educators mentioned during specific questions about FMS. Sarah (T) and Jonny (E) specifically grouped the FMSs into “ball skills”, “balance skills”, and “locomotor skills”, as defined by Gallahue and Donnelly [7], showing the strongest understanding of FMS. Jonny (E) demonstrated further understanding by stating that they are the “basic movements required to complete physical activity”. “Gross and fine motor development” were mentioned by Melissa (T), Karis (H), Brigit (H), and Steven (T) as important elements of the EYFS curriculum and development in EYFS children. The terms “agility”, “balance”, “coordination”, and “speed”, as pointed out by Kirsten (T), Leo (E), and Sarah (T), were more typical of educators who were specialists in PE. The terms “moving and handling”, as mentioned by Melissa (T), are used in the EYFS framework set out by the English government [22]. The framework fails to highlight FMSs development during the EYFS, meaning children are not always afforded the chance to learn and develop these skills until key stage 1. This highlights a lack of guidance around physical development and FMS; future research and government policy should focus on creating a better framework for educators delivering PE at the EYFS.

Educators were given the opportunity to express their thoughts on the importance of PE and FMSs in the EYFS and if they felt more emphasis on physical skills and development were needed: “I think they have to be taught, and certain fundamental movement skills more so than others. So, balance and linking movements and movement skills, you will develop naturally over time, but I do think there has to be some element of teaching in there as well. Whereas ball skills, if there’s no exposure to or teaching to that, then I don’t think learning will take place as naturally” Jonny (E). This supports the view that locomotor skills are performed with higher competency at earlier ages than ball skills, as reported in recent reviews [25,58]. Teaching FMSs through approaches such as implementing simple learning cues and using skill questions has been successful in previous work by Foweather and colleagues [59]. “Every child is individual”, as said by Lorna (T), was a concern for most, relating to how some children would need more tuition than others. This was commonly related to parental involvement, as “you don’t always have the enthusiastic parents that want to teach them” Ruth (E), meaning children may have not been encouraged to develop these skills from an early age. These responses highlight the need for adaptability and avoiding a “one-size-fits-all” approach to the tuition of FMSs at the EYFS. Future interventions and frameworks must have greater consideration for this factor.

### 3.5. Intervention Experience, Needs, and Training Delivery

All educators demonstrated awareness of interventions and many already had these in place in their school: “we had smart moves [a gross motor development intervention] as an intervention” Lorna (T), or “we do an intervention every morning” Melissa (T), and “we’ll get people coming in and leading these interventions and it’s great for those six weeks” Brigit (H). Some educators had experience of intervention delivery during their role, “I manage the smart moves programme that we run in primary schools” Jonny (E). These data show that educational settings are important places for accessible interventions for children.

Educators were asked what would make delivery and training easier with a novel intervention. “A day’s worth of training” Ruth (E), and “I think visually seeing [the intervention]” Jonny (E), with “someone delivering it first for you to watch as an example of it” Leah (T), and “taught like the staff were children” Caitlin (T). Interviewees stated that they found these techniques were the best way to learn about specific intervention delivery and ensure readiness to deliver themselves. Having “learning outcomes are a big one…understanding what the kids are trying to achieve” Kristen (T) “with a lesson plan” Ruth(E) were important. While others expressed the need for “the equipment or access to a computer programme or the notes” Karis(H), as well as “a clear expectation of what resources would be required…depending on cost” Melissa (T).

Some educators also mentioned the need for the intervention to be adaptable or have a “framework and scaffold, some sort of structure” Leah (T) for staff to work from and develop individually for their classes and schools. “Rather than bespoke lesson plans, because every class, every child is different…maybe an overarching view of their skill development and progression of skills document” Sarah (T), so “if you planned it together [with the teacher] and made it bespoke and then left things, that it wouldn’t just finish, so there’s some longevity in it” Brigit (H). Finally, educators were keen to recognise “if we can teach everyone at the same time it’s inclusive” Sarah (T), including “autistic children, your children with disabilities, inclusive games with them” Steven (T) it could increase the success of the intervention. Developing sessions to cater for all children is therefore of great importance to the educators of EYFS children and something which should be promoted more.

All educators stated that they would be confident in delivery of a new intervention or confident of the staff within their schools for intervention delivery: “we do have really good teaching assistants at the school, where there are interventions, they’re able to follow those instructions” Lorna (T). Educators are keen to implement change in their school environments, provide new experiences for their pupils and develop themselves as educators. “I think people would welcome that [interventions] in schools a lot, because it is an area people are probably weaker on” Sarah (T), and “for me to do it [the intervention] and get feedback” Melissa (T). Previous work by Lawless et al., [21], concluded that more confident and knowledgeable staff were needed to implement interventions in primary education. Suggestions by our educators for suitable training opportunities are potentially low cost and achievable, given the availability and accessibility of digital platforms.

Educators wanted to develop their teaching practices and underpin their approaches by using relevant and recent research: “through my experience in elite sport and other stuff that I work in, scientifically backed evidence is far more prevalent, so why should it not be the case within grassroots or within physical education” Jonny (E). However, it is “more effort than it’s worth for most…to find the relevant research. So, it definitely needs to be more accessible” Kristen (T), making sure “it can be short and sharp information, and ‘try this’, rather than feeling like you’ve got to go back to a journal to read it” Brigit (H). These views are a reminder for researchers to make their research as accessible as possible, so it can be understood by those who are most important. Stakeholder views are not only important for the design and delivery of future interventions [15] but also for mainstream lessons for EYFS and PE.

### 3.6. Limitations and Strengths

Although this study recruited a sample from around England, contribution was lacking from areas in the North of England. The North of England has some of the most deprived areas in England [31]. Additionally, all participants were Caucasian and predominantly female. Although this is reflective of educators in England [60], it would have been beneficial to gain perspectives from more male educators and individuals of different ethnic backgrounds, particularly for schools of different faith and religion, as it is recognised children from minority ethnic backgrounds partake in less PA [2]. Finally, this study was conducted during the COVID-19 pandemic, so with the reported decreases in PA in English children [61], it could be possible that educators were paying more attention to increasing PA and PE opportunities at school; however, educators were asked to discuss the pre-COVID practices. A notable strength of this study is the recognition of a wide range of factors affecting children’s education and how these have been perceived by influential educators at the heart of the English school system, through helpful and insightful accounts of implementation, including what works well and why, as well as considerations for intervention design and delivery.

## 4. Conclusions

This study demonstrates the perceptions and values that educators have surrounding PE and PA within EYFS school settings. This study highlights significant multi-faceted barriers that educators face when aiding children to perform adequate PA, which include a lack confidence to teach PE and FMSs and reduced parental involvement. These results show that, to improve PA, the quality of PE, and FMSs tuition in the EYFS, intervention, training, and resources are required. Consideration to the cost of these activities is important, as is parental education, especially in areas of deprivation. Additionally, the format of intervention delivery and training should consider factors including outdoor delivery, remote and video training, and increasing skills educators believe require more tuition, such as object control. Notably, this study collected important stakeholder views, thoughts, and opinions on how best to increase PA and deliver interventions within school environments, which is a novel contribution to research.

## Figures and Tables

**Table 1:** Summary of themes and key quotes.

Theme		
Physical activity and physical education in school settings for EYFS ^1^	Opportunities for PA ^2^	“break times, lunchtimes and PE ^3^ and play opportunities” Jonny (E). “There’s lots and lots of opportunities for outdoor play, and there’s equipment that’s rotated around the week” Lorna (T)
	Extra-curricular	“numerous different football clubs for boys and girls, multisport clubs, dance, and gymnastics that happen after school” Caitlin (T)
Benefits of PE and PA for young children	Confidence	“good self-esteem, so just being able to do those fundamental movement skills, it gives them so much confidence, so that they feel competent in something” Kristen (T)
	Excelling outside the classroom	“because there’s more to you than the calculations you can do in your head or how well you can speak a different language” Melissa (T)
The barriers and challenges to achieving sufficient PA/PE for children faced by educators, parents, and children	Teacher knowledge	“Unless they’ve [EYFS teachers] got a PE background they lack the confidence to teach something like that (PE)” Caitlin (T)
	Sedentary Lifestyles	“kids have maybe got a tablet, and you don’t move on a tablet, do you? It’s just the way people parent has changed hasn’t it, because life changed” Leah (T)
	Deprivation	“can’t afford to take them to do extra-curricular things, so they tend to sit in front of a TV all of the time…even just going for a walk and climbing, things like that, they don’t do” Caitlin (T)
	Parent beliefs	“She turns around to me and says, ‘my mum says I don’t need friends and I don’t need sport to get into Oxford’” Kristen (T)
Educator knowledge of fundamental movement skills and key opportunities for development	Basic FMSs ^4^	“ball skills”, “balance skills”, and “locomotor skills” Sarah (T) and Jonny (E) “Running, jumping, hopping, throwing, skipping, and catching” were skills that eight educators mentioned.
	EYFS framework	“moving and handling” Melissa (T)
	Parent support	“you don’t always have the enthusiastic parents that want to teach them” Ruth (E)
Intervention experience, needs, and training delivery	Experience	“we’ll get people coming in and leading these interventions, and it’s great for those six weeks” Brigit (H)
	Training delivery	“someone delivering it first for you to watch as an example of it” Leah (T) “the equipment or access to a computer programme or the notes” Karis (H)
	Intervention elements	“Rather than bespoke lesson plans, because every class, every child is different…maybe an overarching view of their skill development and progression of skills document” Sarah (T) “autistic children, your children with disabilities, inclusive games with them (children)” Steven (T)
	Using relevant research	“it can be short and sharp information, and ‘try this’, rather than feeling like you’ve got to go back to a journal to read it” Brigit (H)

^1^ EYFS = early years foundation stage, ^2^ PA = physical activity, ^3^ PE = physical education, ^4^ FMSs = fundamental movement Skills.

## Data Availability

The data presented in this study are available on request from the corresponding author. The data are not publicly available due to ethical and GDPR reasons.

## Appendix A

**Table A1:** School Area and English Indices Deprivation.

School Area	IoD
Leicestershire	31,479
North London	4121
Warwickshire	30,640
Worcestershire	14,043
Coventry	1832
Gloucestershire	26,172
South-East London	14,786
Coventry	17,711
Worcestershire	20,632
Somerset	29,374

## Appendix B. Interview Schedule: Name of Study: The Value of Physical Education and Activity for Early Years Foundation Stage Children in England

- What does the phrase “fundamental movement skills” mean to you as a practitioner?
  - Are there any particular categories you would split these skills in to?
  - Do you feel your school provides adequate opportunity for children to develop their FMS?
- Do you think physical skills develop as a child matures, or do you think physical skills should be taught?
- Do you feel PE should hold the same value as other academic subjects, especially at EYFS?
  - Do you think PE should be supported by scientific research methods? What do you think the benefits of doing this are?
- Would you feel confident delivering a PE intervention to an EYFS class if training was provided? Why?
  - What would make you more prepared for delivery of these?
- Do you believe the physical activity government guidelines of 180 min per day for children aged 5 years and below are sufficient and achievable? What barrier do you think teachers and schools face to achieving at least 30 min/day of MVPA for children?
  - Within your school environment or the environment that you work in, how do you approach achieving these guidelines?
  - Do you think the approach could be improved?
- Do you think there are benefits to children being physically active?
  - What do you feel children achieve from being physically active?
- Do you notice any key differences between children who are more physically capable than others?
  - Physically, socially, academically?
- With a lack of research around stability and balance skills at the ages of 4 to 5 years old in the UK, how important do you think these skills are? How would you work on these with EYFS students?
  - What sort of tasks do you think balance and stability aid within the school environment and in PE?
